# Buffalo Medical and Surgical Association

**Published:** 1885-11

**Authors:** 


### Buffalo Medical and Surgical Association.

*Meeting of October 6, 1885.*

The President, Dr. C. G. Stockton, in the chair; Dr. F. R. Campbell, Secretary. Present—Drs. Bartlett, Grosvenor, Moody, Frederick, W. W. Potter, Eli Long, B. G. Long, A. A. Hubbell, Wm. Ring, Jr., Brecht, Van Peyma, Park and Broardt.

The minutes of the last meeting were read and approved.

A motion to postpone the reading of Dr. Frederick's paper to some future meeting, when there would be a larger attendance, was made by Dr. W. W. Potter, and carried, and the President called for voluntary communications.

At this time, several gentlemen came into the room, and a reconsideration of the motion to postpone the reading of the essay was moved and carried.

Dr. C. C. Frederick, the essayist of the evening, read a paper, entitled, " Epithelioma Uteri, Complicating Labor." (Published in this number of the JOURNAL).

*Discussion.*—Dr. W. W. Potter complimented the essayist upon the manner in which he had presented the subject, and especially upon the treatment of the case, which he considered the best that could have been adopted. Such cases as these show that conception may take place in spite of extensive uterine disease. The only criticism he would offer upon the treatment was, the solution of bichloride of mercury employed (1 to 2000) was too strong. A weaker one would, in his opinion, have answered every purpose. In operating upon these cases, the galvano-cautery should be used when only one lip of the cervix is affected; but where the disease is more extensive, he would prefer other methods of operation by which the diseased tissue could be more thoroughly removed.

Dr. Grosvenor remarked that the operation for the removal of the diseased tissue should be deferred until the latter months of pregnancy, as the time for recurrence of the disease would thus be diminished, and the cicatrix formed after the operation would be less indurated.

Dr. Bartlett stated that he had never seen a case similar to the one reported by Dr. Frederick in an extensive obstetric practice of twenty-five years, and considered the essayist fortunate in meeting with one so early in his career. He believed this to be the first case reported to the Association since its establishment. He would not recommend opium to protract a labor, as in the majority of cases he had found that this drug increased the pains. He considered opium a good remedy for nervous irritability in labor.

Dr. Van Peyma spoke of the difficulty of diagnosing these cases. He had seen a case where the cervix of a pregnant uterus was indurated, the first stage of labor very protracted, and malignant disease suspected. But the subsequent history of the case demonstrated that the disease was not cancerous. He would have considered the paper more valuable if the writer had tabulated the results of cases collected.

Dr. Mary Moody asked about the color of the cervix, the time that elapsed between delivery and the beginning of the fever, and the amount of tissue lost.

Dr. Stockton mentioned a case of this sort, recently reported by Dr. Jewett, of Brooklyn, in which Cæsarian section was performed, being the first case of Cæsarian section for epithelioma uteri performed in this country.

Dr. Frederick stated, in conclusion, that the color of the os was red, oozing blood, and that there were shreds of sphacelated tissue hanging from it. The fever appeared on the second day after delivery.

*Voluntary Communications.*—Dr. Roswell Park reported a case of lipoma of the spermatic cord recently removed by him. The patient was a German, 45 years of age. There was a swelling in the right side of the scrotum, half the size of a child's head. At first, Dr. Park suspected hydrocele, but on introducing the trochar and canula, no fluid was obtained. The sheath of the cord was then opened, and a fatty tumor, weighing three pounds, was enucleated. Small accumulations of fat are frequently found in the situation, but a tumor of this size is rarely seen.

Dr. Bartlett reported the case of a night watchman, who was attacked with headache and fever, in this city. As he became no better during three weeks, he went to his home in the country, where he had well-marked typhoid fever. His wife, who, in the meantime, had been confined, was taken with the same disease, and a brother had the same symptoms. The sanitary arrangements of the house and grounds were almost faultless, and he could discover nothing about the place which could produce the disease. He thought the case showed that typhoid fever was directly infectious or contagious.

The meeting then adjourned.

